# Niche Divergence in the South American Freshwater Crustacean *Aegla* Provides Insights Into Its Diversification

**DOI:** 10.1002/ece3.74344

**Published:** 2026-09-11

**Authors:** Gislaine Puli, Álvaro Augusto Mainardi, Héllen Sbruzzi, Sandro Santos, Marlise Ladvocat Bartholomei‐Santos

**Affiliations:** ^1^ Programa de Pós‐graduação em Biodiversidade Animal, Centro de Ciências Naturais e Exatas, Universidade Federal de Santa Maria Santa Maria Rio Grande do Sul Brazil

**Keywords:** Aeglidae, ecological niche, freshwater Decapoda, niche divergence, niche overlap

## Abstract

Understanding how taxa diverge within their ecological niches is essential for revealing mechanisms driving biodiversity and biogeographic patterns, particularly in regions with complex environmental gradients. Ecological niche dynamics have profound implications for speciation and species distributions, yet remain underexplored in freshwater organisms. Here, we investigated ecological niche variation among clades of *Aegla* (Aeglidae), a diverse freshwater crustacean genus endemic to southern South America, to uncover links between niche differentiation and lineage diversification. Using MaxEnt to build niche models with climatic and elevation variables, we assessed niche overlap and similarity in environmental space via PCA‐env, Hellinger's *I*, and Schoener's *D* metrics, complemented by equivalence and similarity tests. We found pronounced niche divergence among *Aegla* clades, characterized by distinct climatic preferences and low‐to‐moderate pairwise overlap. Niche equivalence tests rejected conservatism in all comparisons. This divergence is consistent with the combined effects of geographical isolation among drainage basins and regional climatic heterogeneity, which may have restricted dispersal and promoted the independent occupation of contrasting environmental conditions. These patterns suggest that ecological differentiation, together with historical geographic isolation, is closely associated with diversification within the group. Our results demonstrate that ecological adaptation has likely contributed to diversification in *Aegla,* highlighting niche divergence as an important component of freshwater biodiversity. These insights emphasize the sensitivity of such taxa to ongoing environmental changes and advance our understanding of ecological processes in shaping evolutionary patterns in South American aquatic systems.

## Graphical abstract

We investigated niche divergence among *Aegla* clades of freshwater crustaceans from southern South America. Results show distinct climatic preferences with low overlap, rejecting conservatism and linking ecology to diversification, highlighting niche divergence driving freshwater biodiversity.

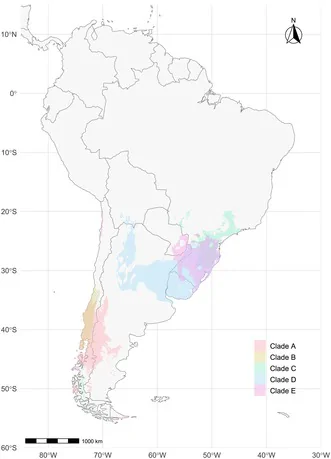

## 1 Introduction

Biological diversity and its geographic distribution around the globe are the result of a complex evolutionary trajectory, whose underlying patterns and mechanisms continue to constitute one of the main focuses of research in evolutionary biology and biogeography. In this field, ecological factors have received increasing attention, with a rising number of studies using the dynamics of niche evolution to elucidate the factors driving lineage diversification and speciation (Peterson 2011; Wooten and Gibbs 2012; Cuervo et al. 2021; Vaissi 2022; Padilha et al. 2025). Diversification events then also began to be associated with niche conservatism, characterized by the maintenance of ancestral ecological characteristics and high niche overlap between related taxa (Cuervo et al. 2021; Wiens and Graham 2005), as well as niche divergence, which involves adaptation to different environments and promotes ecological differentiation and diversification even among phylogenetically close taxa (Cuervo et al. 2021; Dormann et al. 2010; Wooten and Gibbs 2012).

Investigating ecological niche similarity and divergence among related species provides valuable insights into the processes that shape evolutionary diversification and geographic distribution. Niche conservation among closely related species suggests a predominant role for geographic isolation in diversification, while divergence patterns indicate a greater influence of ecological factors on evolutionary trajectories (Knouft et al. 2006; Peterson et al. 1999; Wooten and Gibbs 2012). This dynamic is particularly evident in biogeographically complex regions, such as South America, where habitat diversity can favor adaptation to different niches and contribute to high levels of biodiversity (Avise et al. 1987; Myers et al. 2000; Morrone 2004, 2006). This scenario makes the South American continent attractive for investigations that integrate ecological, evolutionary, and spatial aspects to unravel diversity patterns.

Endemic to southern South America, the family Aeglidae is notable for bringing together freshwater crustaceans currently represented by a single extant genus, *Aegla*, which is highly diverse and comprises about 100 described species (Arantes et al. 2024, 2025; Bueno et al. 2024; Colavite et al. 2024). This family is widely distributed in the southern portion of the continent and, unlike other anomuran crustaceans (such as hermit crabs), has a unique evolutionary history marked by a transition from marine to continental habitats (Bartholomei‐Santos et al. 2020; Pérez‐Losada et al. 2004), as evidenced by the existence of two fossil marine genera (Feldmann 1984; Feldmann et al. 1998). Extant aeglids include both widely distributed and restricted‐range species of *Aegla*, grouped into five phylogenetic clades, and exhibiting patterns of genetic structure throughout their geographic range (Bartholomei‐Santos et al. 2020; Pérez‐Losada et al. 2004). Although advances have been made in understanding the diversity and evolutionary history of the group (Bartholomei‐Santos et al. 2020; Pérez‐Losada et al. 2004; Puli et al. 2025), the influence of environmental factors on the differentiation and maintenance of these clades remains poorly explored. Furthermore, the diversity of occupied habitats (rivers, streams, lakes, and caves) and the fact that approximately 70% of the species are threatened with extinction (Boos et al. 2016; Santos et al. 2017) reinforce the importance of the group as a model for studies on diversification and conservation in environmentally complex regions.

In this study, we investigated whether the five *Aegla* clades (A, B, C, D, and E, as defined by Pérez‐Losada et al. 2004) exhibit similarity or differentiation in their ecological niches. To this end, we constructed ecological niche models for each clade based on large‐scale climatic variables and georeferenced occurrence records. We then assessed whether the environments occupied by these clades are more similar or more distinct than expected under a null model. When integrated with previously established phylogeographic relationships for the group (Pérez‐Losada et al. 2004; Bartholomei‐Santos et al. 2020), these results will contribute to a comprehensive perspective on the climatic niche dynamics among *Aegla* clades, deepening our understanding of the ecological factors that influence the diversification of this ecologically unique group.

## 2 Materials and Methods

### 2.1 Species Occurrence Data

**FIGURE 1:**
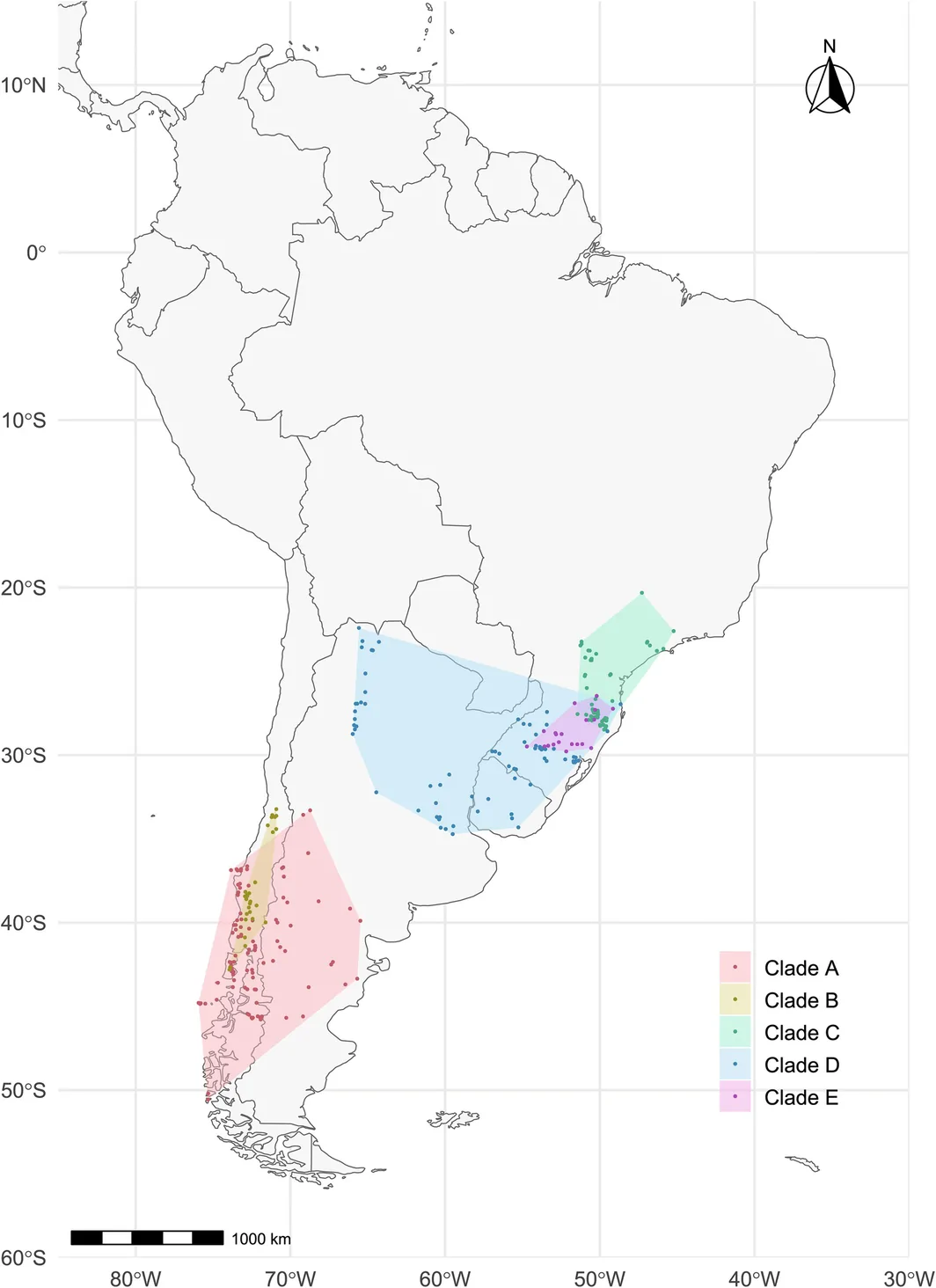
Map of southern South America showing the location of *Aegla* clades and the filtered occurrence points used in this study. WGS84 map projection.

Species occurrence data were obtained from the published literature, compiling information from 35 peer‐reviewed studies. The database of compiled occurrence records and respective references is available in Table S1 (Supporting Information S1). The five phylogenetic clades used as analytical units (A‐E) were originally recovered by Pérez‐Losada et al. (2004) from molecular phylogenetic analyses of the species recognized at that time. Subsequently described species were assigned to these clades only when their placement had been established by molecular evidence in later studies (Table S1). Because cryptic species complexes are common in *Aegla* (Crivellaro et al. 2018; Moraes et al. 2016; Zimmermann et al. 2021), and only some putative species within these complexes have been phylogenetically assessed, we did not assign taxa to clades based solely on morphology or geographic occurrence. We therefore included occurrence records only for species with molecularly supported clade assignments. Using the A‐E clades as analytical units allowed us to compare ecological niche dynamics among lineages with distinct evolutionary histories while minimizing misclassification of occurrence records (Figure S1). The database was filtered to correct georeferencing errors and remove duplicate localities using the R package “CoordinateCleaner.” To minimize spatial autocorrelation in subsequent analyses, we applied a minimum distance filter of 5 km between records using the R package “spThin.” Our final georeferenced species occurrence dataset included 98 occurrence records for clade A, 28 for clade B, 59 for clade C, 74 for clade D, and 20 for clade E (Figure 1).

### 2.2 Environmental Data and Variable Selection

We obtained the bioclimatic and elevation variables from the WorldClim v. 2.1 database (Fick and Hijmans 2017), with a spatial resolution of 2.5 arcminutes (~5 km^2^). The data were then cropped into the geographic area of interest using the R package “raster.” Variable selection was based on a correlation analysis, in which pairs of highly correlated variables (Pearson's correlation coefficient *R* > 0.75) were evaluated, retaining, within each pair, the one considered most ecologically relevant for the genus *Aegla*. As a result, the bioclimatic variables bio1, bio2, bio4, bio9, bio12, bio15, and elevation were selected for the subsequent analyses (Table S2).

### 2.3 Niche Modeling

Ecological niche modeling was performed using the MaxEnt algorithm (Phillips et al. 2019), implemented in the R package “dismo.” Models were generated with 10 bootstrap replicates, using 70% of the occurrence points for calibration and 30% for validation. A maximum of 10,000 background points and 5000 iterations were defined. Model performance was measured using the area under the ROC curve (AUC) and the True Skill Statistic (TSS). The adopted configurations showed superior performance in model evaluation and generated projections consistent with the clade distribution, compared to other parameter combinations tested. Continuous models were converted to binary presence‐absence maps based on the maximum training sensitivity plus specificity metric threshold.

### 2.4 Ecological Niche Conservatism and Niche Divergence

First, we explored the ecological differences between *Aegla* clades based on the environmental variables using the nonparametric Kruskal‐Wallis and Dunn's post hoc tests. Violin plots were employed as a visual tool to represent the distribution and range of variation of each clade for each variable. We also extracted the maximum, mean, and minimum occurrence limits of the clades for each variable. We then applied the PCA‐env approach proposed by Broennimann et al. (2012) to quantify niche overlap along the main environmental axes. To support the interpretation of the results, we used PCA biplots and kernel density plots in environmental space, which allowed us to visualize the distribution of species across the variables considered.

To investigate the hypotheses of niche equivalence and similarity among *Aegla* clades, we used the distance overlap metrics Hellinger's *I* and Schoener's *D*, implemented in the R package “ecospat” (Broennimann et al. 2012; Warren et al. 2008). While the niche equivalence test assesses whether two taxa occupy environmentally indistinguishable niches through the random reallocation of occurrences between areas, the similarity test evaluates whether the observed overlap between their niches differs from that expected when comparing a real niche with simulated random niches in the other taxon's area (Broennimann et al. 2012). Because the niche similarity test is directional, each comparison pair was evaluated in both directions, resulting in two tests per pair.

For each comparison, niche equivalence and similarity tests were run with 1000 permutations, generating a null distribution of the overlap metrics (*I* and *D*). This approach allowed us to assess the significance of the results, considering as statistically significant any value observed outside the 95% range of the simulated values. To investigate different response patterns among the clades, we adopted two criteria in the tests: the “higher” argument was used to detect evidence of niche conservation (when overlap is greater than expected by chance), while the “lower” argument was applied to identify possible signs of niche divergence (when overlap is less than expected). The interpretation of overlap values followed the descriptive categories proposed by Rödder and Engler (2011), as follows: 0–0.2 = absent or very low overlap; 0.2–0.4 = low; 0.4–0.6 = moderate; 0.6–0.8 = high; and 0.8–1.0 = very high.

All analyses were conducted in R Statistical Software 4.5.1 (R Core Team 2025), using RStudio 3.6.0.

## 3 Results

**FIGURE 2:**
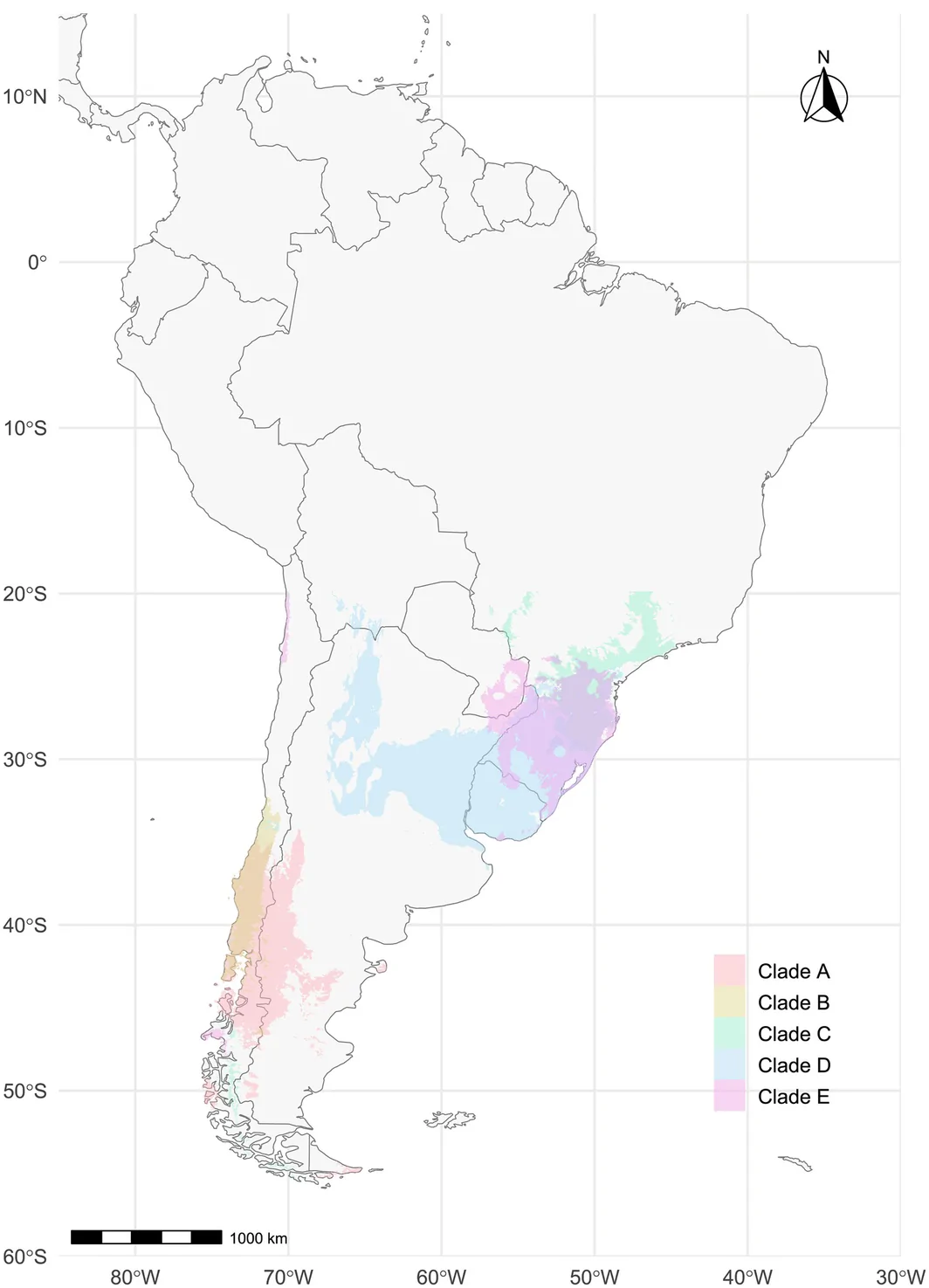
Projected environmental suitability for the *Aegla* clades. WGS84 map projection.

The ENMs showed good predictive capacity, with average AUC and TSS of 0.96 and 0.79 for clade A, 0.99 and 0.89 for B, 0.98 and 0.93 for C, 0.93 and 0.63 for D, and 0.98 and 0.84 for E. Predictions of habitat suitability were consistent with the geographic distribution of the clades: A and B in Chile and southern Argentina, clade D in northern Argentina, Uruguay, and Brazil, and C and E in southeastern and southern Brazil (Figure 2).

**FIGURE 3:**
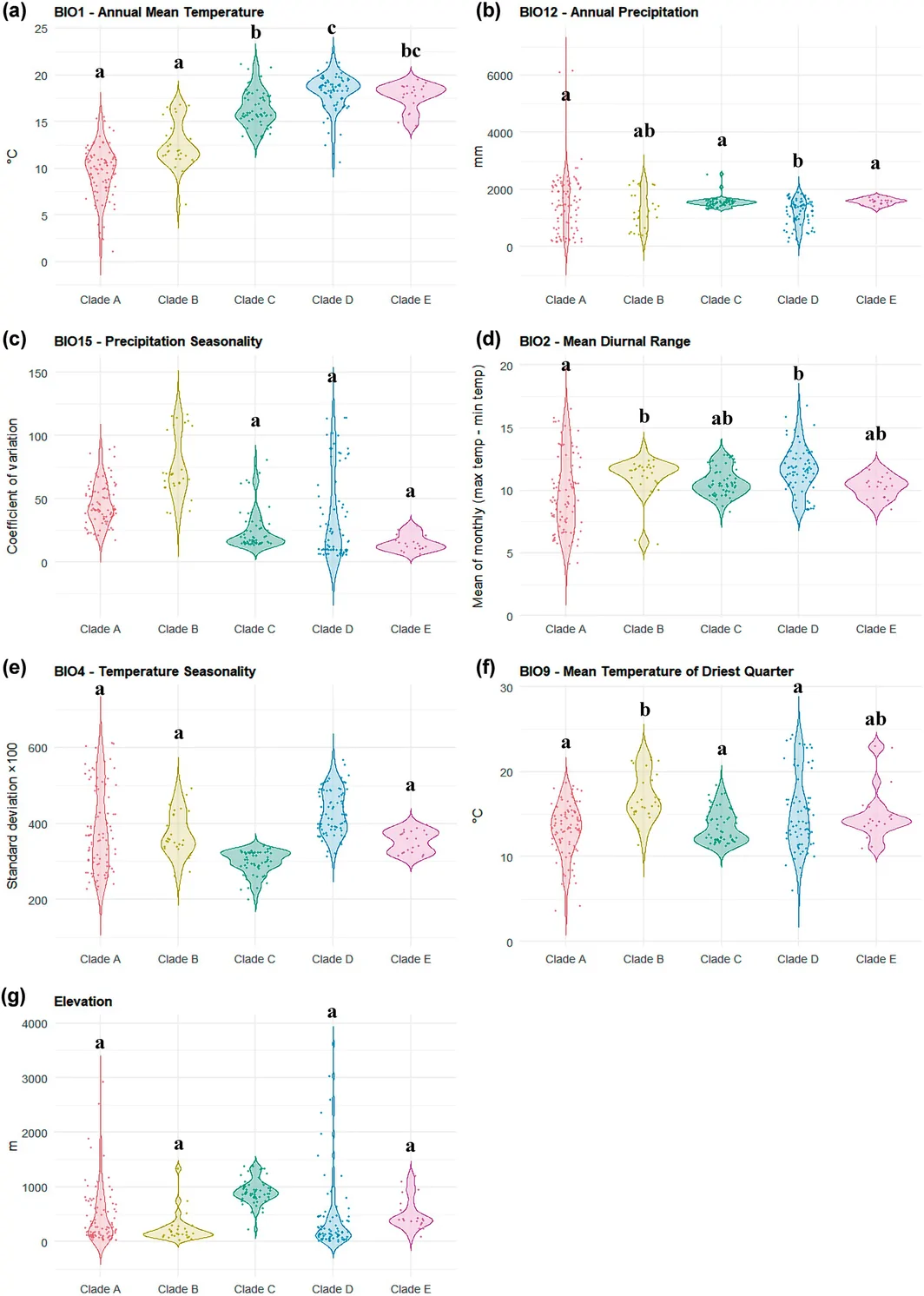
Violin plots of the selected bioclimatic variables, based on values extracted from occurrence records of each *Aegla* clade. Equal letters denote pairs of clades with non‐significant differences after Kruskal‐Wallis and Dunn's post hoc tests. (a) Annual Mean Temperature, (b) Annual Precipitation, (c) Precipitation Seasonality, (d) Mean Diurnal Range, (e) Temperature Seasonality, (f) Mean Temperature of Driest Quarter, (g) Elevation.

**FIGURE 4:**
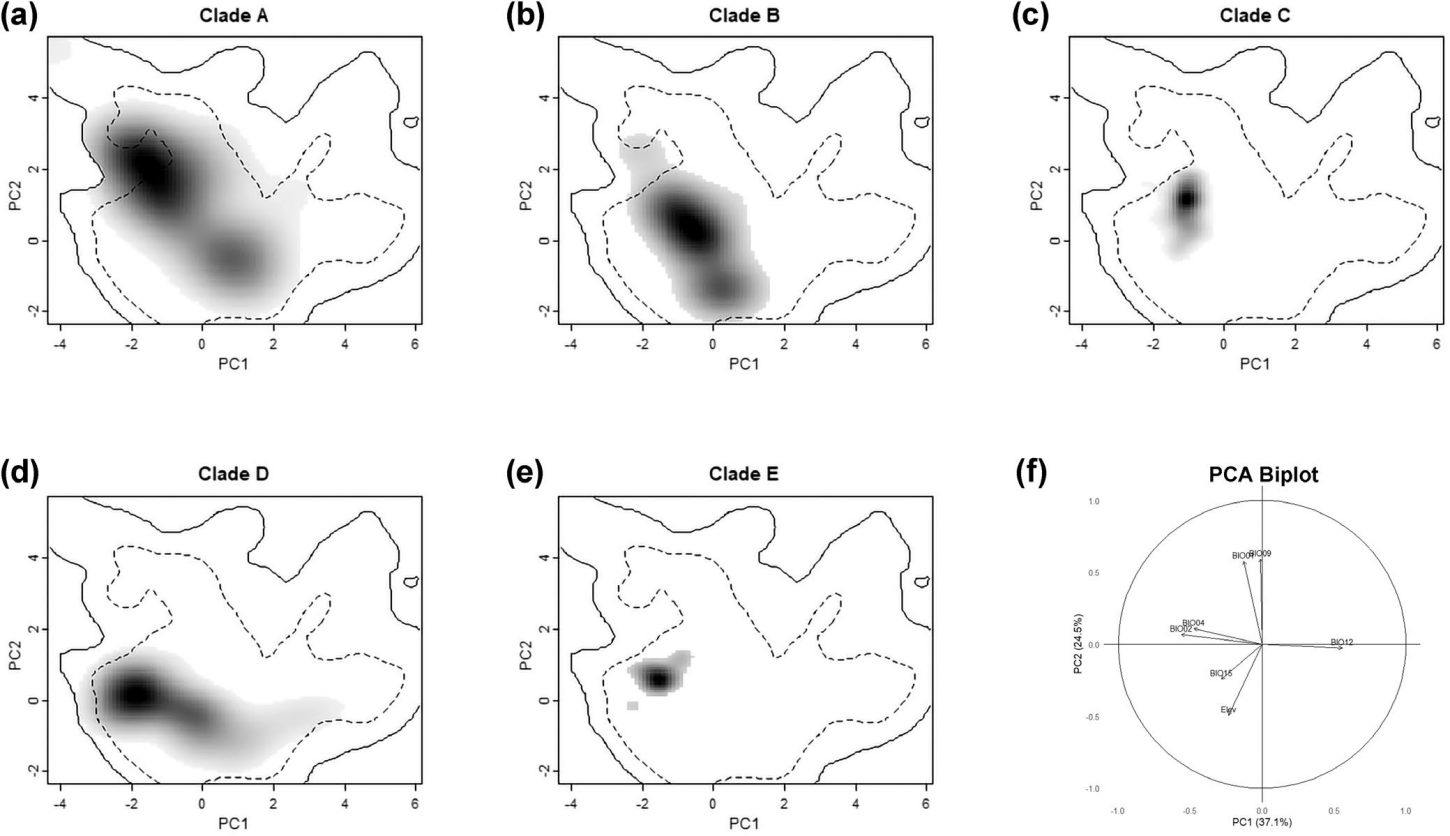
Climatic niches in the southern South America of the clades of *Aegla* in the niche space spanned by two PCA‐axes. The environment available and the environment occupied (realized niche) by each *Aegla* clade are shown, (a) clade A, (b) clade B, (c) clade C, (d) clade D, and (e) clade E. Gray shading represents the density of the occurrences by cell, with darker shading indicating higher density of occurrences. Solid contour and dashed lines visualize 100% and 50% of the available environment (background), respectively. (f) The PCA biplot represents the contribution of the seven bioclimatic variables on the two PCA‐axes: Bio1—Annual Mean Temperature, bio2—Mean Diurnal Range, bio4—Temperature Seasonality, bio9—Mean Temperature of Driest Quarter, bio12—Annual Precipitation, bio15—Precipitation Seasonality, and elevation.

The Kruskal‐Wallis analyses, occurrence thresholds for each environmental variable, and kernel density plots in environmental space show variations in climatic conditions associated with the different clades, with distinctions in the climatic niches occupied by each (Figures 3 and 4; Table S3). The first two axes of the PCA explain 61.6% of the variation in the data. The first PCA axis is positively related to bio12 (Annual Precipitation) and negatively related to bio4 (Temperature Seasonality) and bio2 (Mean Diurnal Range). The second PCA axis is positively related to bio9 (Mean Temperature of Driest Quarter) and bio1 (Annual Mean Temperature) and negatively related to elevation (Figure 4).

The results of niche overlaps with Hellinger's *I* metric showed that clades A‐B, B‐C, and C‐E present moderate overlaps (0.4–0.6), while the remaining pairs presented low to very limited overlap (< 0.4) (Table 1; Figures S2 and S3). However, the niche equivalence test did not support conservatism in any of the cases, while niche divergence is supported in all the comparisons (Table 1). In the cases of B‐C, B‐E, and D‐E, the occupied niches were ecologically distinct but more similar to each other than expected by chance (Table 1). The Schoener's *D* metric showed low to very limited overlap (< 0.4) for all clade pairs (Table S4).

**TABLE 1:** Hellinger's *I* and *p*‐values for the equivalence and similarity tests for niche divergence (D) and conservatism (C) hypotheses among the *Aegla* clades.

Clades pairs	Hellinger's *I*	Equivalency *p*‐values	Similarity *p*‐values Clade1 ➔ Clade 2	Similarity *p*‐values Clade2 ➔ Clade1
A‐B	0.533	**0.001** (D)	0.173	0.171
A‐C	0.284	**0.001** (D)	0.139	0.132
A‐D	0.150	**0.001** (D)	0.376	0.377
A‐E	0.118	**0.001** (D)	0.756	0.759
B‐C	0.536	**0.004** (D)	**0.033** (C)	**0.030** (C)
B‐D	0.290	**0.001** (D)	0.252	0.252
B‐E	0.317	**0.001** (D)	**0.025** (C)	**0.034** (C)
C‐D	0.171	**0.001** (D)	0.760	0.751
C‐E	0.437	**0.001** (D)	0.065	0.060
D‐E	0.286	**0.001** (D)	**0.024** (C)	**0.027** (C)

*Note:* Significant *p*‐values (boldface).

## 4 Discussion

Understanding the similarity characteristics and degree of ecological niche divergence between closely related taxa is fundamental to clarifying the relative contribution of ecological factors to species diversification processes (Broennimann et al. 2012; Wiens et al. 2010). This study provides a comprehensive assessment of the ecological niches of *Aegla* clades, a highly endemic and threatened genus of freshwater crustaceans. In particular, we investigated whether the clades maintain a similar ecological niche (conservatism) or occupy distinct niches (divergence). The most striking result of this study was the absence of ecological niche conservatism in all comparisons tested, even in cases where the niches were more similar than expected by chance (B‐C, B‐E, and D‐E), indicating that the *Aegla* clades have followed distinct ecological trajectories. Because *Aegla* is restricted to freshwater habitats and has limited opportunities for dispersal across drainage divides, these distinct trajectories are likely to have developed within a spatially fragmented landscape in which geographic isolation and environmental differentiation may have acted jointly (Barber et al. 2012; Loretán et al. 2020; Pérez‐Losada et al. 2004). In general, the environmental suitability estimates projected by the ENMs strongly corresponded with the known ranges of occurrence for the *Aegla* clades, reflecting the geographic patterns previously described for the group (Pérez‐Losada et al. 2004; Bartholomei‐Santos et al. 2020). Thus, the areas of environmental suitability remained restricted to the regions currently occupied by each clade, without indicating significant expansions into adjacent areas. This pattern suggests a possible combination of ecological specialization and historical limitations on dispersal and occupation (Barber et al. 2012; Pérez‐Losada et al. 2004; Puli et al. 2025; Xu et al. 2009). For freshwater lineages, drainage divides, topographic barriers, and the discontinuous distribution of suitable stream habitats can limit connectivity among populations, promoting lineage isolation and the subsequent accumulation of genetic, morphological, and ecological differences (Barber et al. 2012; Loretán et al. 2020; Pérez‐Losada et al. 2004; Dias et al. 2013).

The cohesive spatial configuration observed, combined with the divergence in occupied ecological niches, reinforces the hypothesis that diversification in *Aegla* is associated with the occupation of distinct environmental settings. Although present‐day ENMs do not directly demonstrate long‐term geographic stability, the restricted suitability patterns are consistent with persistent spatial structuring among clades. This interpretation is consistent with evidence from *A. singularis* , in which populations from the Paraná and Uruguay river basins show genetic and morphological differentiation associated with restricted gene flow across the Sierra Central Mountains (Loretán et al. 2020). It is also consistent with the phylogeographic history of *A. neuquensis* , for which fragmentation between the Negro and Chico‐Chubut river systems has been linked to the disappearance of headwater paleolakes during Patagonian glaciation (Barber et al. 2012).

We observed consistent evidence of niche differentiation among clades, supported by Kruskal‐Wallis tests and comparisons in environmental space. The hypothesis that pairs of clades occupy identical environmental niches was rejected in all cases by niche equivalence tests, and most background similarity tests indicated that the observed degree of overlap is consistent with that expected by chance (Table 1), reinforcing the pattern of divergence. Only pairs B‐C, B‐E, and D‐E showed niche similarity greater than expected by chance in the directional similarity tests. However, because niche equivalence was rejected in all comparisons, these results indicate partial environmental similarity rather than identical or unequivocally conserved niches. These clades share similar environmental characteristics in some variables (Figure 3). Greater‐than‐expected niche similarity does not necessarily reflect evolutionary conservatism or spatial proximity; instead, it may arise from convergent environmental associations or similar ecological constraints influencing niche occupation in separate regions. More broadly, the rejection of niche equivalence despite similarity in some comparisons suggests that clades may retain responses to comparable environmental conditions while occupying non‐identical portions of environmental space. Such a pattern is compatible with a history in which isolation among drainage systems preceded, or occurred concurrently with, ecological differentiation (Barber et al. 2012; Loretán et al. 2020; Pérez‐Losada et al. 2004; Warren et al. 2008).

Our results showed that *Aegla* clades respond to different environmental conditions and that ecological divergence may have cooperated with lineage differentiation within the group, reflecting distinct environmental associations and contributing to the group's diversification. However, ecological niche models describe associations between present‐day occurrences and environmental conditions; therefore, they cannot by themselves distinguish whether niche divergence was a direct cause of lineage divergence, a consequence of allopatric isolation, or the outcome of both processes acting together (Soberón 2007; Peterson et al. 2011). The pattern found is consistent with previous studies that highlight the central role of ecological divergence in speciation and diversification for different taxa (Cuervo et al. 2021; Vaissi 2022; Wooten and Gibbs 2012), reinforcing the idea that divergent environmental adaptations can be important determinants in the generation and maintenance of biodiversity. In *Aegla*, this process should be interpreted within a historical biogeographic framework in which geographic isolation among river basins, topographic barriers, and changes in hydrological connectivity may have generated opportunities for independent ecological trajectories (Barber et al. 2012; Loretán et al. 2020; Pérez‐Losada et al. 2004).

Furthermore, variations in niche overlap and environmental preferences were not shown to be related to phylogenetic relationships between clades, a pattern already observed in other biological groups (Cuervo et al. 2021; Vaissi 2022; Vaissi and Rezaei 2022; Wooten and Gibbs 2012), which suggests that local ecological factors may act independently of evolutionary history in niche delimitation. Alternatively, the lack of a simple correspondence between phylogenetic proximity and niche similarity may reflect the fact that clades with different evolutionary histories have been exposed to distinct regional climatic regimes and hydrological landscapes (Barber et al. 2012; Mejía et al. 2022; Pérez‐Losada et al. 2004). It is worth noting that clades A and B, the first to diverge in the *Aegla* phylogeny and occupying overlapping distributions in the southern portion of the genus range (Pérez‐Losada et al. 2004), presented a moderate niche overlap and no evidence of niche conservatism. Their niche divergence suggests that spatial overlap alone does not imply ecological equivalence, particularly where clades occupy different portions of climatic, elevational, or hydrological environmental space. On the other hand, the weakly supported clades D and E, the last ones to branch off, inhabiting adjacent areas (Pérez‐Losada et al. 2004), presented low niche overlap, with no niche equivalence but more similarity than randomly expected. Together, these contrasting patterns indicate that niche divergence within *Aegla* cannot be explained solely by phylogenetic distance or geographic proximity, but likely reflects the interaction between lineage history, geographic isolation, and environmental heterogeneity (Barber et al. 2012; Mejía et al. 2022; Loretán et al. 2020; Pérez‐Losada et al. 2004).

Finally, we suggest that the high species diversification observed in *Aegla* can be explained, at least in part, by the ecological niche differentiation observed among the clades in our study. Rather than operating in isolation, niche differentiation likely interacted with the fragmented nature of South American freshwater systems, in which drainage reorganization, topographic barriers, and past climatic fluctuations may have repeatedly altered opportunities for dispersal, isolation, and persistence (Barber et al. 2012; Loretán et al. 2020; Pérez‐Losada et al. 2004; Puli et al. 2025). These findings reinforce the role of ecological divergence as an important mechanism in generating diversity in freshwater environments and highlight the importance of integrating ecological and evolutionary approaches to understand diversification patterns in widely distributed groups. This integrative perspective is also critical for conservation, as projections for some aeglid species indicate that climatic change may differentially affect environmental suitability and genetic diversity, potentially increasing the vulnerability of lineages restricted to fragmented freshwater habitats (Puli et al. 2026). Future studies combining genetic, physiological, and environmental data could deepen our understanding of the processes shaping the evolutionary history of *Aegla* and similar groups, as well as their vulnerability to the rapid environmental changes affecting global biodiversity.

## Author Contributions

**Gislaine Puli:** conceptualization (equal), formal analysis (equal), investigation (equal), visualization (equal), writing – original draft (lead). **Álvaro Augusto Mainardi:** conceptualization (equal), formal analysis (equal), investigation (equal), visualization (equal), writing – review and editing (supporting). **Héllen Sbruzzi:** formal analysis (equal), investigation (equal), writing – review and editing (supporting). **Sandro Santos:** conceptualization (supporting), project administration (equal), resources (equal), supervision (equal), writing – review and editing (equal). **Marlise Ladvocat Bartholomei‐Santos:** conceptualization (equal), project administration (equal), resources (equal), supervision (equal), writing – review and editing (equal).

## Funding

This work was supported by Conselho Nacional de Desenvolvimento Científico e Tecnológico (308894/2025‐1, 311690/2018‐1). Coordenação de Aperfeiçoamento de Pessoal de Nível Superior.

## Conflicts of Interest

The authors declare no conflicts of interest.

## Supporting information


**Figure S1:** Schematic representation of the genus *Aegla*'s phylogeny, with the clade names and species belonging to each clade according to Pérez‐Losada et al. (2004). The cladogram does not include new species or cryptic complexes identified after 2004.
**Figure S2:** Histograms show the observed niche overlap (Hellinger's distance *I*) between the *Aegla* clades (bars with a diamond) and simulated niche overlaps (gray bars) on which tests of niche equivalency and niche similarity are calculated. The significance of the tests is shown in Table 1.
**Figure S3:** Visualization of ecological niche dynamics between pairs of *Aegla* clades in environmental space.

**Table S1:** Geographic coordinates of *Aegla* occurrence records compiled in the present study. Clades A to E are defined according to Pérez‐Losada et al. (2004).
**Table S2:** Bioclimatic and elevation variables used in this study.
**Table S3:** Occurrence thresholds of *Aegla* clades for each variable used.
**Table S4:** Schoener's *D* and *p*‐values for the similarity and equivalence tests for niche conservatism (C) and divergence (D) hypotheses among the *Aegla* clades.

## Data Availability

The data that supports the findings of this study are available in the Supporting Information S1 and S2 of this article.
