# Supplementary material for: Niche Divergence in the South American Freshwater Crustacean Aegla Provides Insights Into Its Diversification
Source: Ecol Evol. 2026 Sep 11;16(9):e74344. doi: 10.1002/ece3.74344 (PMC13564243; doi:10.1002/ece3.74344)
Supplement: Supplementary file 1 [file ECE3-16-e74344-s002.pdf]

Supporting Information: Niche divergence in the South American freshwater crustacean *Aegla* provides insights into its diversification

Puli et al. (2026)

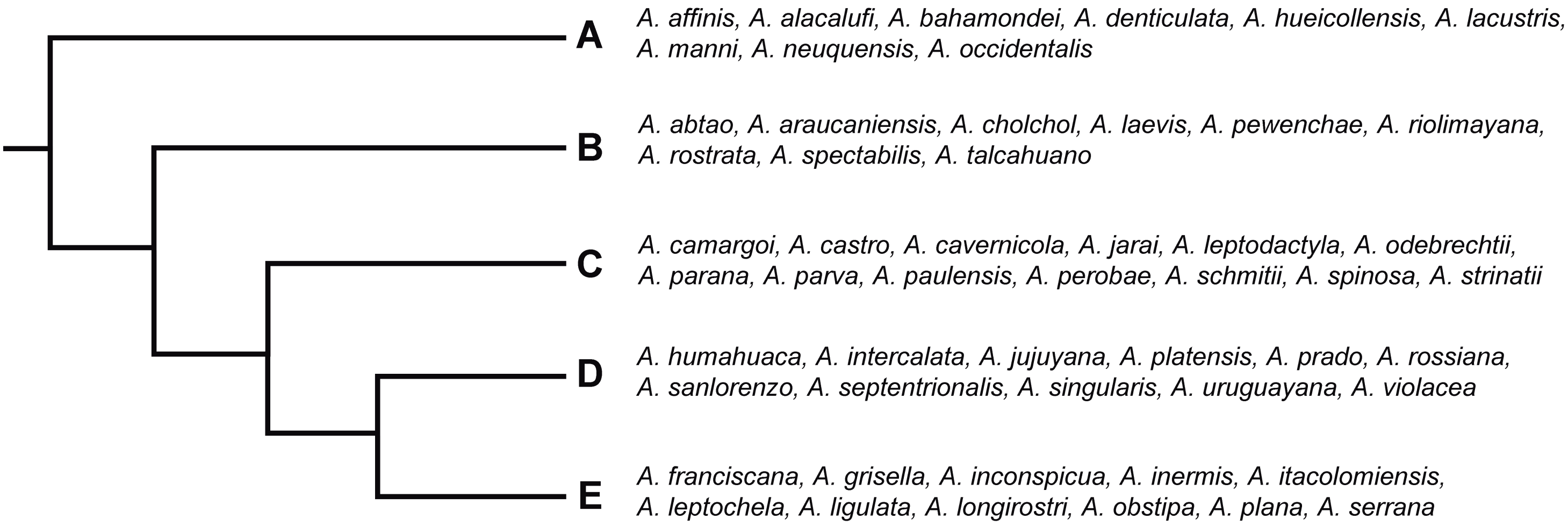

Figure S1 Schematic representation of the genus *Aegla*'s phylogeny, with the clade names and species belonging to each clade according to Pérez-Losada et al. (2004). The cladogram does not include new species or cryptic complexes identified after 2004.

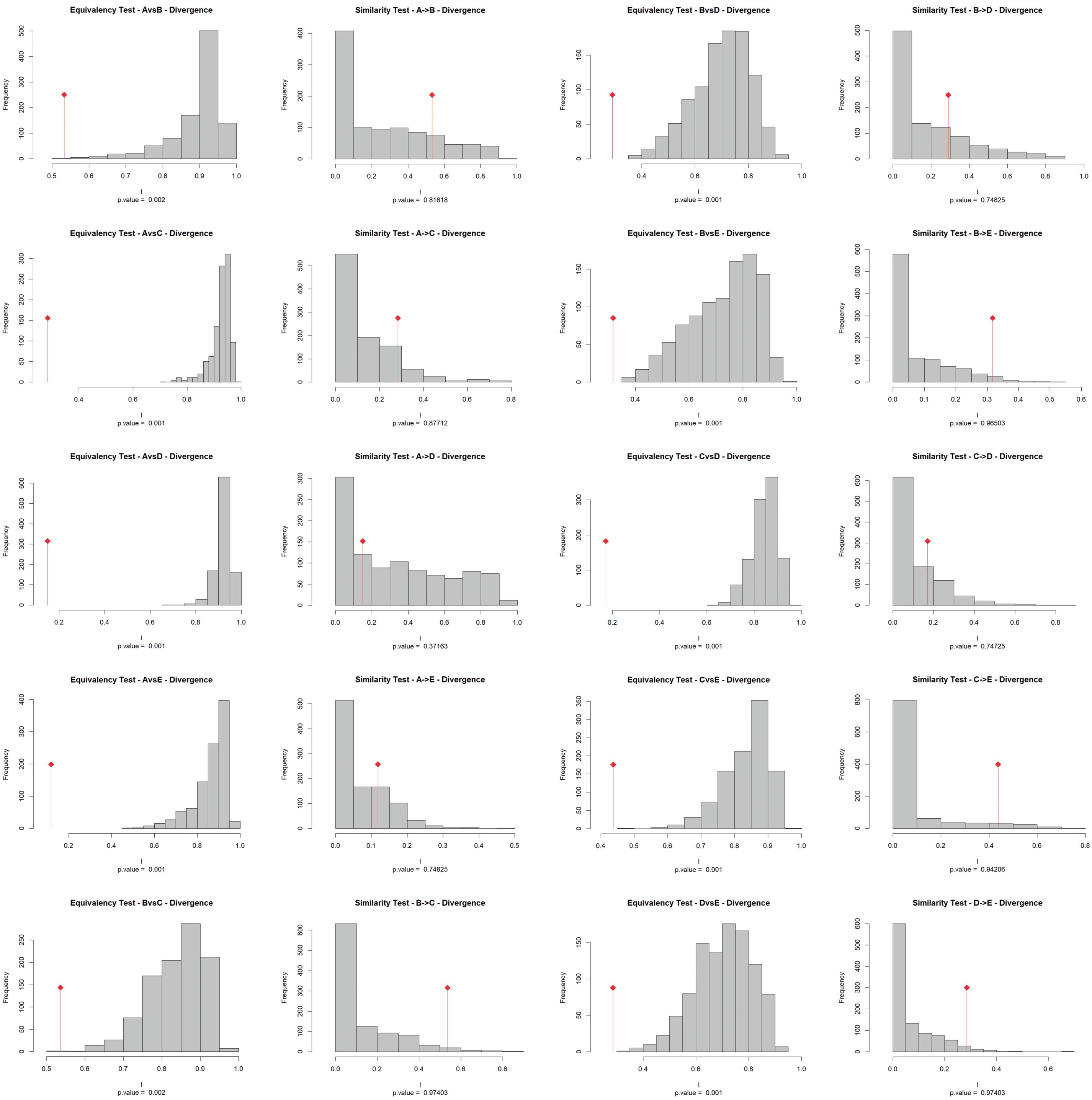

Figure S2 Histograms show the observed niche overlap (Hellinger's distance *I*) between the *Aegla* clades (bars with a diamond) and simulated niche overlaps (grey bars) on which tests of niche equivalency and niche similarity are calculated. The significance of the tests is shown in Table 1.

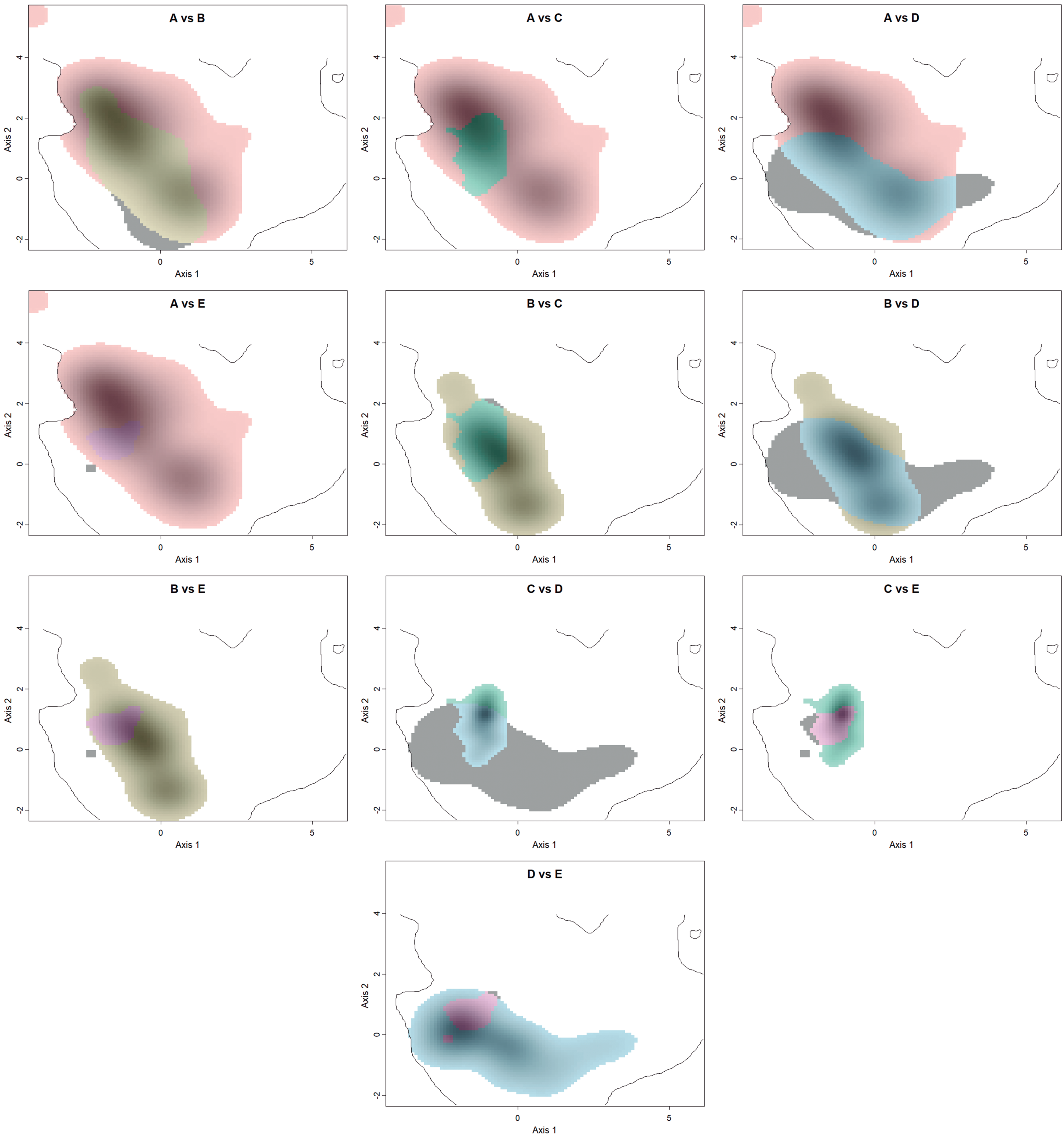

Figure S3 Visualization of ecological niche dynamics between pairs of *Aegla* clades in environmental space.
